# Observations of enhanced rainfall variability in Kenya, East Africa

**DOI:** 10.1038/s41598-024-63786-2

**Published:** 2024-06-05

**Authors:** Susan M. Kotikot, Erica A. H. Smithwick, Helen Greatrex

**Affiliations:** 1 Department of Geography, Earth and Environmental Systems Institute, The Pennsylvania State University, University Park, PA USA; 2 Department of Geography, Department of Statistics, Institute for Computational and Data Sciences, The Pennsylvania State University, University Park, PA USA

**Keywords:** Climate change, Rainfall variability, Self-organizing maps, Wavelet analysis, Kenya, Climate-change mitigation, Climate-change impacts

## Abstract

Understanding local patterns of rainfall variability is of great concern in East Africa, where agricultural productivity is dominantly rainfall dependent. However, East African rainfall climatology is influenced by numerous drivers operating at multiple scales, and local patterns of variability are not adequately understood. Here, we show evidence of substantial variability of local rainfall patterns between 1981 and 2021 at the national and county level in Kenya, East Africa. Results show anomalous patterns of both wetting and drying in both the long and short rainy seasons, with evidence of increased frequency of extreme wet and dry events through time. Observations also indicate that seasonal and intraseasonal variability increased significantly after 2013, coincident with diminished coherence between ENSO (El Nino Southern Oscillation) and rainfall. Increasing frequency and magnitude of rainfall variability suggests increasing need for local-level climate change adaptation strategies.

## Introduction

Climate variability is of great concern for countries whose economies rely on rain-fed agriculture, especially when climate services are inadequate, and adaptive capacity is low. Extreme events such as drought can directly impact the productivity of both crops and pasture systems^1,2^. Thus, understanding local patterns of change is the first critical step in developing context specific and risk-based adaptation strategies. Recent trends indicate a strong increase in the intensity and frequency of maximum temperatures across East Africa^3,4^. Based on global CMIP6 estimates, the intensity and frequency of maximum temperatures and of heavy rainfall is expected to increase^5,6^ across East Africa (EA). While these broad scale climate assessments are important for guiding national climate adaptation action plans, they do not highlight local level spatial–temporal changes that are relevant to agropastoral livelihoods to promote contextualized adaptation. Furthermore, large uncertainties remain regarding the spatial and temporal variability of rainfall events^7^.

Rainfall projections for EA are associated with high uncertainty due to complex climate drivers^8^. For example, the outcomes of large-scale atmospheric circulation systems are influenced by local physical features of the landscape that can produce highly diverse climate conditions within very short distances, and that are difficult to directly link to global circulation systems^9,10^. Despite its equatorial location, the EA region is relatively dry compared to the rest of the equatorial belt^11^. Even so, the precarity of agricultural livelihoods in the region is related more to rainfall variability than to annual total rainfall of the growing season^12^*.* Extreme rainfall in 2019 for example, is believed to have caused desert locust reproduction^13^, while recurrent droughts^14^, have caused devastating famine across EA. Increasing velocities of climate changes^15^ can aggravate land degradation, for example through increased rainfall intensity, floods, drought frequency and intensity. However, fine-grained spatial and temporal patterns of rainfall are not understood at local scales, hindering climate adaptation^16^.

In addition to these factors, EA rainfall is influenced by numerous teleconnections (e.g., El Niño-Southern Oscillation (ENSO), Indian Ocean Dipole (IOD), Madden–Julian Oscillation (MJO)^17^), regional (e.g., topography^18,19^ large water bodies^20–22^), and local (e.g., soil moisture^23^, vegetation) controls on its rainfall variability^18,23^. These factors generate variability in seasonality and rainfall amounts at multiple scales. Specifically, the IOD^24^ and ENSO have been shown to influence extreme events (high rates of rainfall and heat convergence associated with aridity) in EA^25,26^ and are major modes of interannual variability, while the MJO is mainly associated with intraseasonal variability^27^. ENSO and IOD affect EA rainfall by influencing preferred regions of rising and descending moisture and air in the Walker circulation^28,29^. Basically, rainfall is enhanced during El Niño and positive IOD events, and drought is enhanced during La Niña and negative IOD events^30^. Although many IOD events develop independently with separate impacts on regional climate from those of the ENSO related variability, ENSO can enhance IOD variability at interannual time scales^31^. Often, negative IOD events will occur during La Niña and positive events will occur during El Niño^32^. The impact on rainfall amount and variability is greatest when ENSO and IOD are in phase.

Interactions among large-scale (atmospheric and ocean circulation) and regional and local factors produce intricate patterns of rainfall. The Intertropical Convergence Zone (ITCZ) governs the seasonality of EA rainfall^33^. It is a band of low pressure around the Earth and marks the convergence of trade winds near the equator^34^. Drastic shifts in the position of the ITCZ can occur due to local and remote forcings^35–37^ with potential implications for seasonal rainfall patterns in EA. ENSO for example, is known to influence interannual variations in the seasonal cycle of the ITCZ^30^. The complex topography of EA affects rainfall primarily by influencing low-level flow which enhances surface heat flux and convection and initiates high frequency mesoscale and sub-synoptic disturbances^18^. For example, the highlands channel flow creating the southwest monsoon flow and Somali Jet^38^ and the Turkana channel converges flow into a low-level jet that impacts low-level divergence, modulating the diurnal cycle of rainfall^19^. Lee rain shadows also form when highlands block moist air flowing from the Congo basin. Large water bodies assure the presence of moisture, which can intensify local level rains^39^. For example, Lake Victoria influences the diurnal cycle of rainfall largely through interactions between orographic effects of the surrounding topography and easterly flow^20–22^. As a source of evapotranspiration, soil moisture (and vegetation) influences water and energy cycles and therefore can trigger atmospheric feedbacks that impact the climate system^23,40^. Forests are especially known to control rainfall patterns at local to regional scales^41^. Depending on the scales of deforestation, forest loss can impact rainfall by inducing local circulations that enhance rainfall or it can reduce precipitation recycling leading to rainfall reduction as has been shown with the Congo Forest^42^. As a result of these multiscalar factors, the EA rainfall regime is marked with extreme spatial heterogeneity and temporal variability where, in recent years, drought and flood years/seasons have alternated.

Typically, the rainfall regime is bimodal over much of EA, with most of the rain falling in the long rains in March, April, and May (MAM) and less during the short rains in October, November, and December (OND)^43^. Previous work has shown that interannual variability is stronger in OND than MAM at broad scales, but OND rainfall is better understood as it has a strong connection to ENSO and IOD^44,45^, whereas MAM is weakly influenced by sea surface temperature (SST) anomalies^46–49^. Although drivers of MAM variability are less well understood^18^, extreme seasons have occurred over the recent years^50–52^ and there is evidence that MAM rainfall over EA is decreasing, contributing to widespread drought and famine^53^. Some researchers attribute MAM decline to a stronger Indian Ocean branch of the Walker Circulation^54,55^ influenced by the Pacific Ocean^56^. More specifically, interactions between La Nina-like patterns in the eastern Pacific and warm western Pacific SST have been linked to drier than normal MAM including the recent widespread MAM 2022 droughts in EA^57^. According to^46^, recent MAM rainfall decline is likely associated with decadal variability in the Pacific Ocean, specifically the negative phase of the Pacific Decadal Oscillation. Others have found that MAM variability is rather influenced by a combination of factors acting on intraseasonal, interannual, decadal, and multidecadal timescales (e.g.^18,58,59^. Furthermore,^18^ found that each month in MAM exhibited marked differences in terms of character, causal factors, and teleconnections, which indicates intraseasonal contrasts.

In this study, we explore spatio-temporal characteristics of rainfall variability in Kenya, and examine the association of rainfall with ENSO and the IOD mode, two major factors of rainfall variability in EA. We reveal heterogeneous patterns of rainfall anomalies at scales relevant to agropastoral livelihoods in EA. First, we uncover archetypal spatial patterns of seasonal rainfall anomalies and their trends over time using the self-organizing map (SOM)^60^ approach and compare between national and regional levels of analysis to establish the potential for localized assessment. SOM is an ideal tool for identifying physically relevant patterns of variability and allows us to assess changes in the frequency of such patterns. Estimated patterns provide a basis for further investigation of physical driving mechanisms including less understood micro to mesoscale systems. Second, we explore the periodicity of rainfall variability and how that variability is changing with time using the continuous wavelet transform^61^, and delineate between interannual, interseasonal, and intraseasonal time scales of rainfall variability. Third, we determine the correlation in time and frequency space, between rainfall and SST indices (Niño3.4 and the Dipole Mode Index (DMI)), and extract features of localized (in time) coherence and phase relationships. The two complementary approaches used in the study provide robust characterization of rainfall patterns allowing us to infer relative variability at local spatial and temporal scales, and how that variability is changing with time. Understanding time scales of rainfall variability, e.g., intraseasonal patterns, is essential for characterizing patterns of seasonal rainfall distribution that determine water availability to crops. Furthermore, season-to-season dynamics (interseasonal variability) are critical for assessing the vulnerability of pastoral livelihoods that involve seasonal migration based on weather and pasture availability. Our findings are critical for informing policy on adaptation strategies especially for agropastoralists in arid and semi-arid areas where rainfall is a major determinant of productivity.

## Data and methodology

The Climate Hazards group InfraRed Rainfall with Station (CHIRPS) v2.0^62^ dataset was used in this study. It is a quasi-global dataset available at a relatively high spatial resolution of 0.05^0^ and multiple time steps (daily, 5-day) beginning 1981 to present. It is developed based on high-resolution infrared satellite measurements and blended with ground observation station data. It therefore closely matches data that is interpolated gauge observations in areas with a dense network of stations. Generally, agreement between CHIRPS and ground observations varies across space and with time depending on the density of gauge stations. CHIRPS was chosen as the primary rainfall dataset in this study due to both its performance in validation studies, and due to its wide-ranging use in both Kenya drought research and operations (allowing comparison between this study and existing research). At present, over ten research papers have validated CHIRPS across Kenya, with a majority finding excellent correlation against reference gauges^62–69^. In addition, CHIRPS is used operationally in Narok for drought early warning bulletins published by Kenya's National Drought Management Authority^70^; is commonly employed as a benchmark dataset in meteorological research across Kenya^71–75^; and was chosen as the input dataset for a wide range of recent drought-relevant research in Narok and the surrounding regions^76,77^. CHIRPS has also been employed as an input dataset for several trend analyses in the East African region and was found to perform well^17,53,66,78–80^.

However, it is important to note that CHIRPS is still likely to have errors, especially as most validation studies focus on the larger East African region rather than examining local meteorological conditions. For example, several papers focused on Kenya have suggested CHIRPS is likely to underestimate rainfall in high elevation zones, including the mountains in Narok county^81^. Others have suggested that CHIRPS might overestimate rainfall in the nearby Kisumu region along the Western shores of Lake Victoria^82^. In addition,^65^ suggested that CHIRPS had a slight dry bias, although in general performing well. In other regions of the world, CHIRPS has been found to struggle to capture extreme rainfall^83^.

Monthly time series of the Dipole Mode Index (DMI) and Niño3.4 SST anomaly index between 1981 and 2021 were used to assess the relationship between the Indian and Pacific Ocean SST and Kenya rainfall anomalies. DMI indicates the east–west temperature gradient of the tropical Indian Ocean associated with the IOD. It is the difference between the Western Tropical Indian Ocean (WTIO) and Southeastern Tropical Indian Ocean (SETIO) SST anomaly indices which are indicators of surface temperatures in the western and southeastern parts of the tropical Indian ocean. WTIO and SETIO are respectively calculated with SSTs within 50 °E–70 °E, 10 °S–10 °N and 90 °E–110 °E, 10 °S–0°. The Niño3.4 is an SST anomaly index that indicates average SST conditions in the tropical pacific between 170 °W and 120 °W, 5 °S–5 °N. Monthly DMI and Niño3.4 SST anomalies are calculated at NOAA Physical Sciences Laboratory using the HadISST1.1 SST dataset and can be downloaded from https://psl.noaa.gov/gcos_wgsp/Timeseries/.

### Study area characteristics

**Figure 1:**
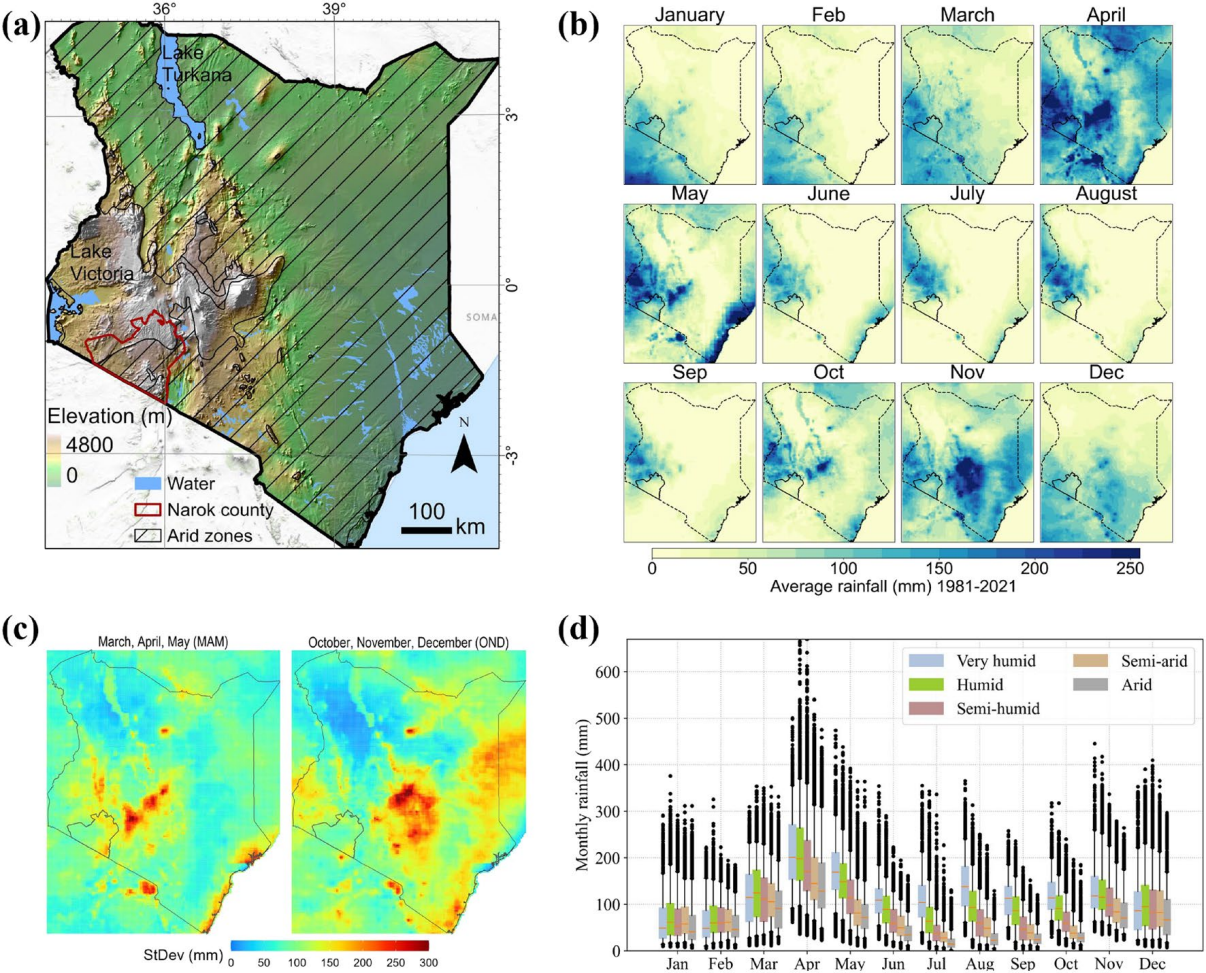
Study area map showing elevation over the geographic extent of Kenya and Narok county (**a**). The arid zone (semi-arid and arid) of the Kenya agroclimatic zones is shown in black hatching. The rest of the area is under the humid (very humid, humid, semi-humid) category. Monthly average (1981–2021) rainfall of Kenya showing spatial heterogeneity in rainfall (**b**). Standard deviation of MAM and OND total rainfall (1981–2021), showing high deviations in the highland and coastal areas (**c**). Boxplot of monthly rainfall spatially averaged over each agro-climatic zone (**d**). There are high variations among the agro-climatic zones for the months between April and November, and arid zones have very low rainfall between July and October. Figure (**a**) was created using ArcGIS Pro 3.2.0, (**b**), (**c**) and (**d**) were created in Python 3.10.9 using Matplotlib 3.7.0^87^ library (https://ieeexplore.ieee.org/document/4160265).

The study area is Kenya (Fig. 1a), a country in equatorial EA. Like the wider equatorial EA, the rainfall regime is historically bimodal over most areas^43^, although over recent years, the timing of seasonal rainfall has become increasingly unpredictable. The spatial distribution of rainfall is highly heterogeneous and mainly follows patterns of topography (Fig. 1b). March, April, May (MAM) has been the typical growing season^84,85^ but recent trends have indicated delayed onset, early cessation of rains, and prolonged dry periods^9,78^. To determine local level patterns, we compare results between national (country) and regional (county) levels of analysis. Narok county was selected as a regional case study site as it exemplifies a typical agropastoral landscape in EA in that it is highly diverse in climate, topography, land cover, and land uses. It is a county in southern Kenya, ~ 18,000 km^2^ in area. Livelihood forms relate to ecological and climate conditions which follow patterns of elevation, and that are strongly influenced by local topography^86^.

### Spatial patterns of rainfall variability

The Self-organizing maps^60^ approach was used to delineate dominant spatial patterns of rainfall anomalies. They are a type of artificial neural network which allows for the nonlinear unsupervised classification of multivariate data into a finite set of representative patterns. A SOM is made up of several nodes in a two-dimensional grid representing the probability density function of a dataset^88^. The nodes are successively adjusted through an iterative training process, until the map becomes well organized and representative of the data^89–91^. Each data point is then assigned to a node based on its similarity (measured by Euclidean distance) and the node with the smallest distance to the data vector is the best match unit (BMU). Compared to other clustering techniques, SOM has a main advantage in that it does not assume stationarity or orthogonality^88,89,92^ making it more appropriate for analysis of environmental data. SOM has successfully been used in the clustering of rainfall data and found to obtain patterns that are readily interpretable^93,94^. Furthermore, they can discover patterns resulting from non-linear processes, and may help reveal physical mechanisms consistent with the observed patterns. The SOM clustering method delineates groups with similar rainfall characteristics, including time evolving patterns associated with climate change.

In this study, monthly CHIRPS data between 1981 and 2021 for MAM and OND seasons were used to train a SOM for each season. Because we are interested in isolating patterns of rainfall anomalies, standardized anomalies of each MAM and OND month were calculated as the monthly values minus the long-term monthly average and divided by the month standard deviation. The monthly standardized anomalies were then used to create a SOM for each season, while adjusting the SOM size to maximize variance and maintain significance. For each of the seasons, a 3 × 2 SOM size was found to fully represent the distribution of rainfall anomaly patterns resulting in six nodes (patterns). To unravel regional patterns of rainfall anomalies, the analysis was done at both the Kenya and Narok levels, in each case resulting in a 3 × 2 SOM size. From each SOM, specific characteristics including, the dominant patterns, the trends in the frequency of occurrence of the patterns, and the spatial heterogeneity of anomalies were derived and compared between seasons and geographic levels of analysis. A potential weakness of the SOM approach is that many free parameters need to be adjusted. Sensitivity tests were conducted on all the free parameters and the best SOM was chosen that provided a good balance based on three standard measures of SOM quality including the Sammon map, quantization error (QE), and topographic error (TE). The Sammon map approximates the Euclidean distance between SOM nodes. It shows similarity among SOM node maps and how the map is ordered. A good Sammon map should be flat (not folded). QE assesses how related the SOM map nodes are to the input data vector. The range of QE values depends on the input data, but they should be continuously declining with SOM training time. TE calculates the percentage of data vectors whose second-best match node is not a neighboring unit. It assesses whether the SOM map is well-ordered. TE values between 10–15% are acceptable. All (MAM and OND for Kenya and Narok levels) SOM patterns exhibited flat Sammon maps, consistently declining QE values, and TE values that are less than 15%.

### Temporal patterns of rainfall variability

To understand time scales of rainfall variability and how that variability changes temporally, the continuous wavelet transform approach was utilized following procedures outlined in^61^. Wavelet transform is a multiresolution analysis that achieves time–frequency representation of transient patterns in time series data^61^. It is an appropriate technique for analyzing signals that have time (when) and frequency (how often) components, such as rainfall data. In continuous wavelet transform, a wavelet is applied as a band pass filter to the time series and shifted temporally at varying time window lengths which allows for time localization of signal properties. A wavelet is a small oscillation defined by a function with zero mean and localized in both time and frequency^95^. In this analysis, the Morlet wavelet was used because it has been shown to provide better feature extraction and a good balance between time and frequency localizations^95–97^. The Morlet wavelet was applied with a non-dimensional frequency of 6 which satisfies the admissibility condition^95^ and ensures that the wavelet scale is comparable to the Fourier period.

Monthly CHIRPS rainfall data were spatially averaged over two groups of agro-climatic zones (humid and arid) separately for each of the two geographic extents (Kenya and Narok) resulting in four time series of monthly average values between 1981 and 2021. The humid category consisted of the very humid, humid, and semi-humid agro-climate zones, and the arid category consisted of the semi-arid and arid zones^98,99^. Delineation of moisture levels allowed for assessment of relative variability between dry and humid areas as related to climatic vulnerability of agropastoral communities, and the two geographic levels allowed for delineation of local features as they are most relevant for local livelihoods. The monthly time series data were then seasonally adjusted through differencing which involves subtracting the previous observation (in the previous season) from the current observation. The seasonally adjusted values were then standardized by subtracting the mean and dividing by the standard deviation to allow comparison between power spectra. Continuous wavelet transforms of the resulting time series data were then performed. For easy comparison between power spectra, the power spectra were normalized by 1/σ^2 to give power relative to that of white noise. The normalized power spectrum can be interpreted as the variance of a time series at a specific period and specific time. The statistical significance of the power spectrum was evaluated relative to the null hypothesis that the signal is generated by a stationary process with lag-1 (AR1) red noise spectrum. To understand fluctuations in power at different scales, scale averaged power spectrum was calculated at predetermined scales of 2–4, 5–10, and 18–30 months respectively defined here as intraseasonal, interseasonal, and interannual timescales. The scale averaged power shows average rainfall variance over time at the specified frequency (scale) bands.

### Influence of large-scale teleconnections

To understand the relationship between rainfall and oceanic SST indices, wavelet coherence between the time series was computed. Wavelet coherence is a measure of the coherence in time–frequency space between power spectra of two time series^61,100^. It can be interpreted as a localized correlation between two time series in time–frequency space and identifies areas in time–frequency space where there is coherence. We calculate the statistical significance of the wavelet coherence using Monte Carlo methods following^100^. The significance is estimated against AR1 red noise as it has been shown that the impact of AR1 coefficients on significance is low. First, we calculated wavelet coherence between time series of Niño3.4 and DMI SST to explore the relationship between them. Wavelet coherence was then calculated between monthly time series of rainfall and both the Niño3.4 and DMI SST indices to determine possible influence of the SST on rainfall. All the data were first standardized before computation.

## Results

### Spatial patterns of rainfall variability

**Figure 2:**
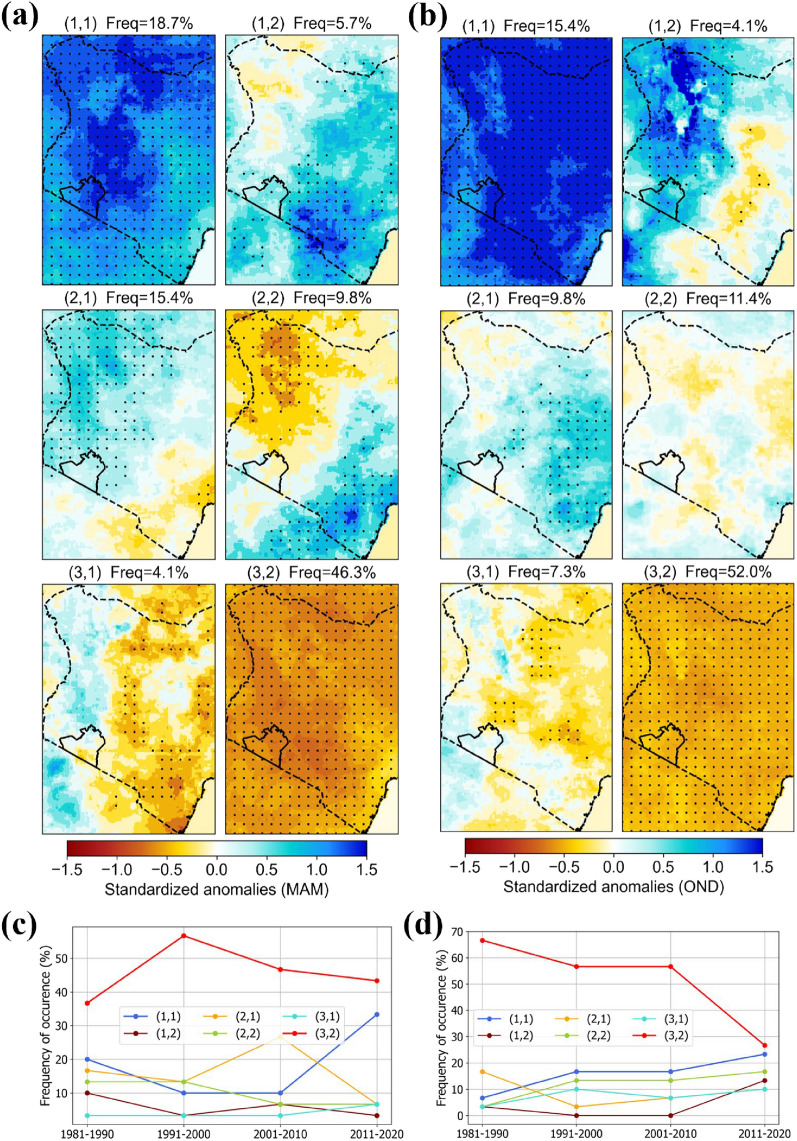
SOM patterns of MAM (**a**) and OND (**b**) rainfall anomalies showing spatial coherence and heterogeneity across Kenya. Patterns are named in order of their position in the 3 by 2 SOM node grid. The percentages show relative frequency of occurrence of each pattern between 1981 and 2021, and stippling shows > 95% significance. Trend in frequency of occurrence of the SOM patterns showing the proportion of MAM (**c**) and OND (**d**) months that the specific pattern was observed within every 10 years period. SOM analysis was done using Matlab R2023b SOM toolbox. Figures were created in Python 3.10.9 using Matplotlib 3.7.0^87^ library (https://ieeexplore.ieee.org/document/4160265).

Results show SOM patterns associated with positive and negative rainfall anomalies across Kenya for both MAM (Fig. 2a) and OND (Fig. 2b). For each season, the six nodes represent archetypal spatial patterns of rainfall anomalies observed between 1981 and 2021. The nodes correspond to the most occurring spatial patterns as indicated by their relative frequencies (shown on the figure). The patterns show areas with coherent rainfall characteristics, and we can infer potential driving factors and potential impacts on associated ecosystems where similar adaptation strategies may be recommended. For both seasons, there are two dominant nodes that show strong positive (1,1) and negative (3,2) anomalies indicating a dry and wet pattern. The dry pattern occurs more frequently compared to the wet pattern which means that most of the MAM and OND months between 1981 and 2021 had lower than average rainfall. For MAM, both nodes (dry and wet patterns) exhibit an increasing trend in their frequency of occurrence over time (Fig. 2c). The increasing trends show that MAM is increasingly characterized by more months of higher than average and lower than average rainfall, and fewer months of average rainfall. For OND, the dry pattern shows a deceasing trend in the frequency of occurrence, and the wet pattern shows an increasing trend (Fig. 2d). These trends imply that OND is increasingly characterized by more months of higher-than-average rainfall and less months of lower-than-average rainfall.

At the national level, there appears to be significant spatial heterogeneity of rainfall anomalies. Within the dominant nodes (1,1 and 3,2), there are spatial differences in the strength of negative and positive anomalies. Other nodes, (1,2), (2,1), (2,2), (3,1) show patterns with both negative and positive anomalies where one part of the country is always dryer (wetter) than average at the same time when other parts are wetter (dryer) than average. Such recurring patterns indicate a likely influence of underlying physical mechanisms including mesoscale to synoptic factors acting and interacting at multiple scales to generate the rainfall patterns. The patterns therefore provide a basis for further investigation into driving factors, and ultimately better understanding of rainfall climatology.

**Figure 3:**
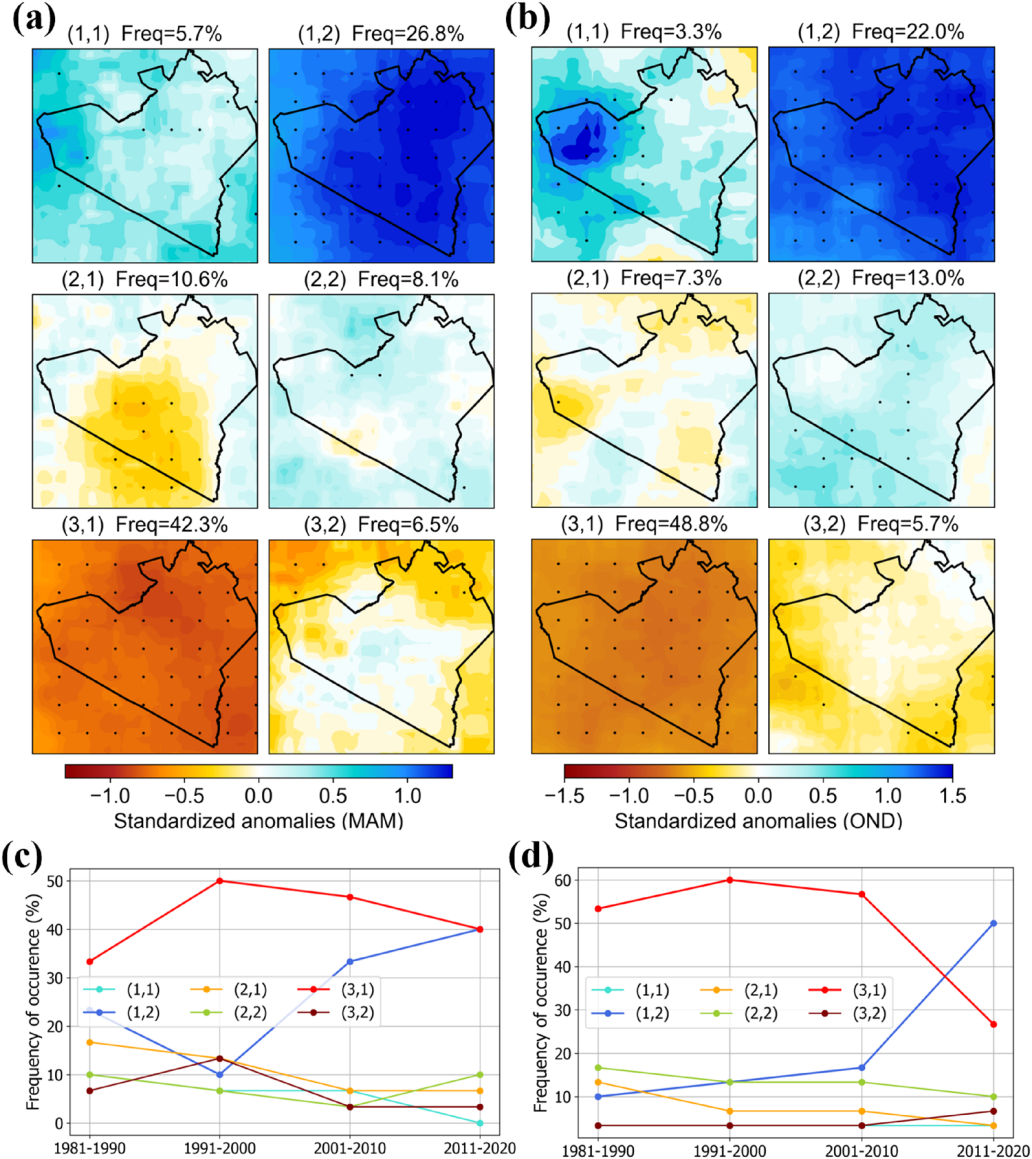
SOM patterns of MAM (**a**) and OND (**b**) rainfall anomalies showing spatial coherence and heterogeneity across Narok county. Patterns are named in order of their position in the 3 by 2 SOM node grid. The percentages show relative frequency of occurrence of each pattern between 1981 and 2021, and stippling shows > 95% significance. Trend in frequency of occurrence of the SOM patterns showing the proportion of MAM (**c**) and OND (**d**) months that the specific pattern was observed within every 10 years period. SOM analysis was done using Matlab R2023b SOM toolbox. Figures were created in Python 3.10.9 using Matplotlib 3.7.0^87^ library (https://ieeexplore.ieee.org/document/4160265).

Nested patterns were observed between the national (Kenya) and regional (Narok) levels of analyses for both MAM and OND. For example, matching node distribution, the dominant patterns, and their trends of occurrence (Fig. 3). At the Narok level, the spatial patterns are accentuated allowing us to see spatial difference at more localized scales. Slight differences in the frequencies of individual patterns between Narok and Kenya are expected because SOM training is an iterative process seeded with random data. However, the overall similarity of patterns between the Narok-level and the Kenya-level demonstrates the replicability of the SOM approach and shows that in both cases, the SOM was well organized.

### Temporal patterns of rainfall variability

**Figure 4:**
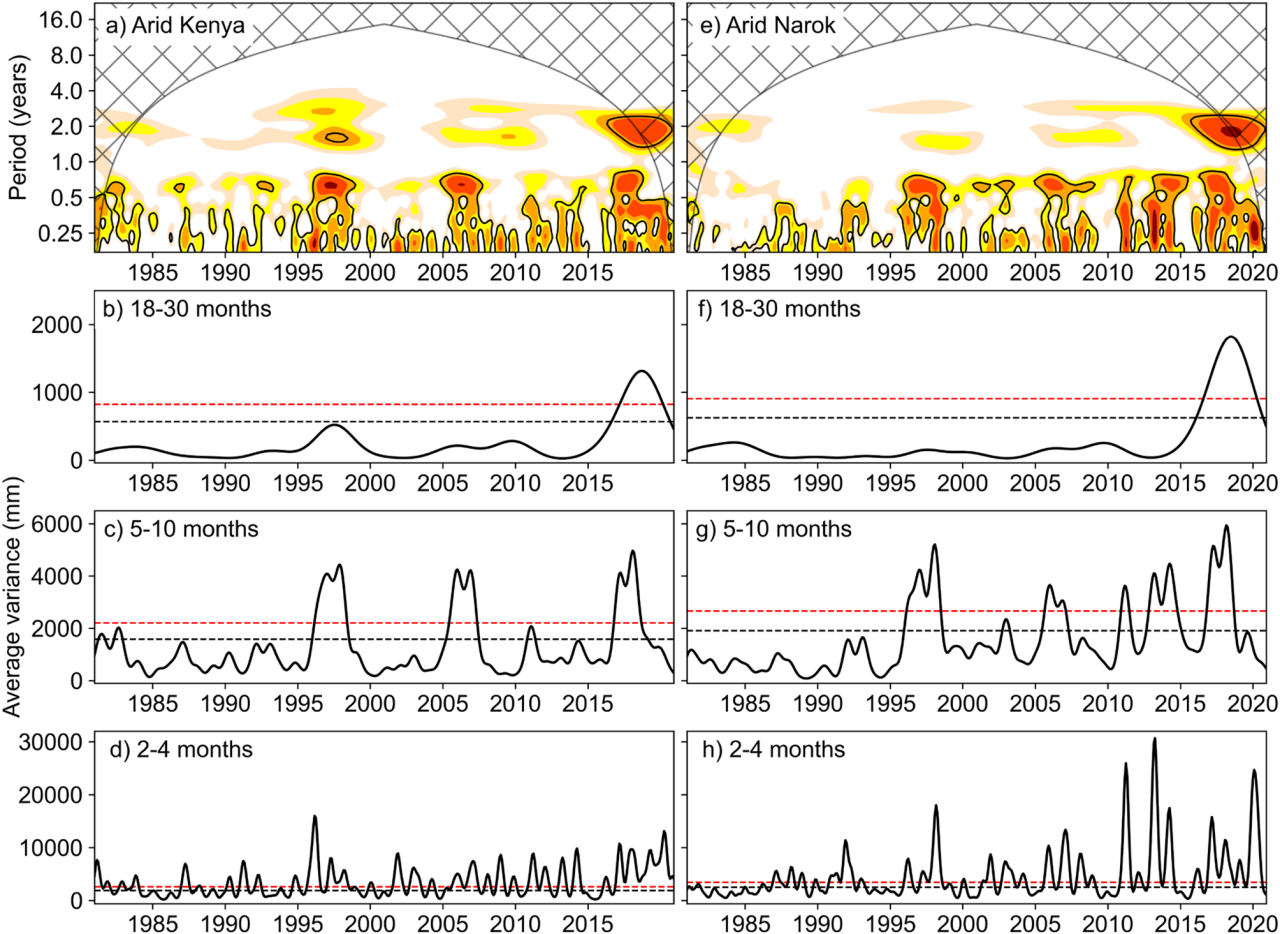
Wavelet power spectra of spatially averaged rainfall over arid agro-climatic zones of Kenya (**a**) and Narok (**e**). In (**a**) and (**e**), color contours represent wavelet power normalized by the squared standard deviation and are at 0, 1, 2, 3, 4 from the lightest to the darkest colors, solid black contours enclose areas of > 0.95 significance relative to a lag-1 red noise spectrum, hatched areas represent the cone of influence—area of the wavelet spectrum where edge effects (errors) due to a finite time series cannot be ignored. Figures (**b**), (**c**), (**d**) and (**f**), (**g**), (**h**), respectively show scale averaged power over the periods 18–30, 5–10, and 2–4 months for (**a**) and (**e**), and show average rainfall variance for those periods. Dotted horizontal lines show the > 0.95 (black) and > 0.99 (red) significance levels. Curves above the dotted lines have significant variance. The power spectra in (**a**) and (**e**) were generated using pyCWT^101^ Python library for spectral analysis. All the figures were created in Python 3.10.9 using Matplotlib 3.7.0^87^ library (https://ieeexplore.ieee.org/document/4160265).

Wavelet power spectra show for arid areas, significant (95% confidence level) rainfall variability at periods (timescales) between 2 and 10 months and, in the recent years (2017–2021), at periods between 18 and 30 months (Fig. 4). Scale-averaged power at predefined periods of 18–30 (Fig. 4b,f), 5–10 (Fig. 4c,g), and 2–4 (Fig. 4d,h) months are defined as interannual, interseasonal and intraseasonal timescales, and are shown for both the national (Kenya) and regional (Narok) levels.

Rainfall periodicity showed different patterns between the national and regional levels. For Kenya, there are episodes of interannual variability between 1997 and 1999 and of interseasonal variability before 1997 that are not evident at the Narok level. At the Narok level, we detect episodes (2002–2004, 2012–2017) of significant interseasonal variability that was not significant at the Kenya level. We also note a marked increase in interseasonal and intraseasonal variability after 2012. Across both Kenya and Narok, we observe increased variability during 1997–1998, 2005–2007, 2017–2018 likely partially a response to El Niño and or a positive IOD, that we discuss in the next section. Comparison between power spectra of arid and humid zones for both Kenya and Narok revealed only inconsiderable differences and therefore results are not shown here.

### Influence of large-scale teleconnections

**Figure 5:**
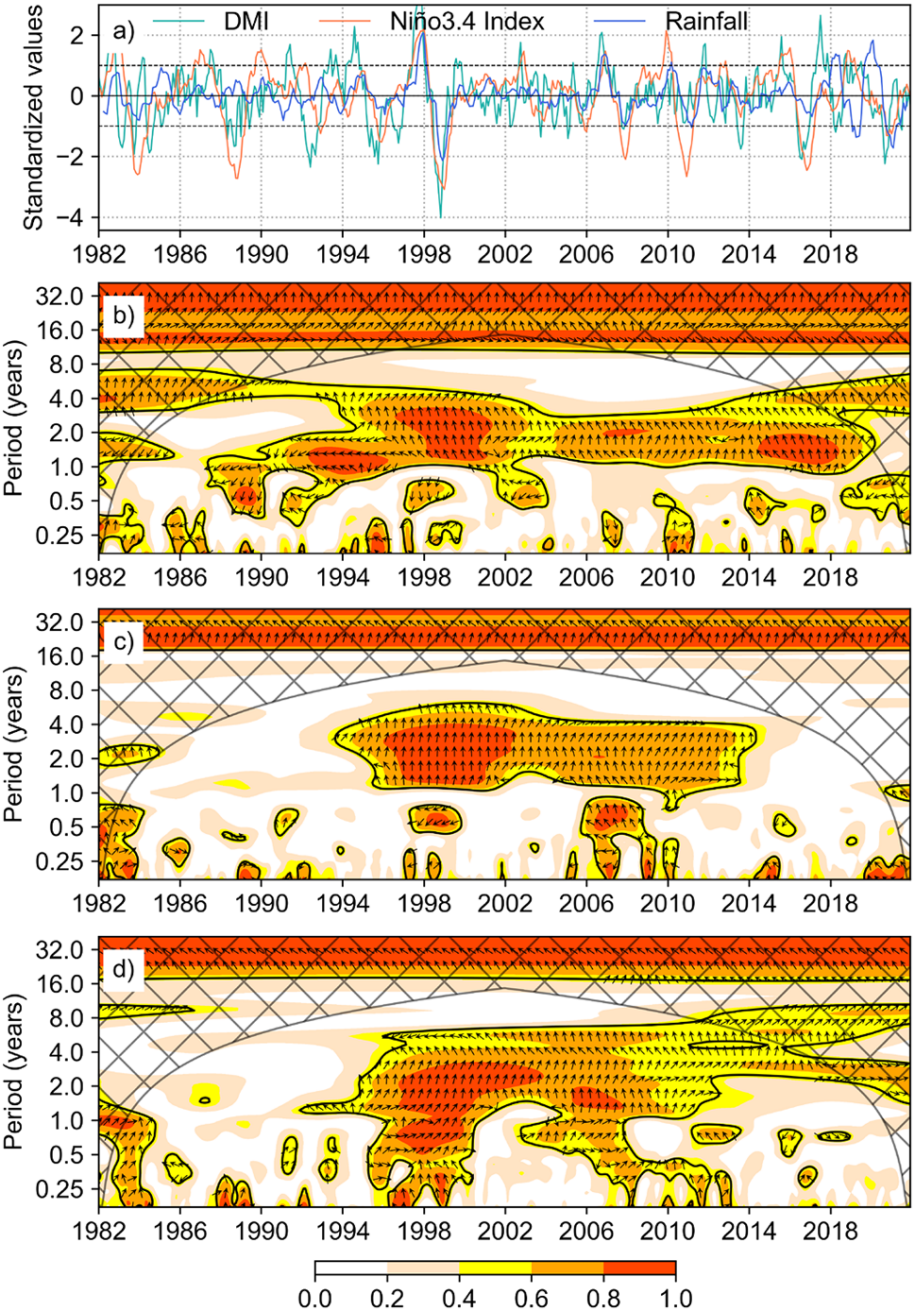
Monthly values of standardized DMI, Standardized Niño 3.4 SST Index, and standardized monthly rainfall (**a**), wavelet coherence between time series of the Niño3.4 SST index and DMI (**b**), Niño 3.4 SST index and rainfall (**c**), DMI and rainfall (**d**). Rainfall data are averages over the Kenya geographic extent. The color scale shows correlation between the time series. Arrows show phase relationships between the time series (in phase if pointing up, anti-phase if pointing down, one signal leading another if pointing right—in this case, Niño 3.4 index leading DMI or Niño 3.4 index leading rainfall or DMI leading rainfall). Wavelet coherence was performed in Python 3.7.0 using pyCWT^101^ library for spectral analysis, and the figures were created using Matplotlib 3.7.0^87^ library (https://ieeexplore.ieee.org/document/4160265).

Wavelet coherence between Niño3.4 SST and DMI (Fig. 5b) shows a large area of significant correlation at time scales between 1 and 4 years. Based on the phase angles shown by arrows on Fig. 5b, there is high coherence with in-phase behavior between Niño3.4 and DMI most of the time between 1997–1998, 2005–2007, and 2014–2018, times when rainfall spectra (Fig. 4) show high power, indicating increased variability. Some areas of the spectrum show out of phase behavior, for example at sub-annual time scales between 1988–1990 and between 1997–1999.

Significant correlation between the SST indices (Niño3.4 SST and DMI) and Kenya rainfall is evident. At scales between 1 and 4 years, there is continuous, strong, and significant coherence between Niño3.4 SST and Kenya rainfall between 1994 and 2013 (Fig. 5c). The coherence between DMI and Kenya rainfall extends to 2018 (Fig. 5d). Given the high coherence between the SST indices at annual time scales between 2014 and 2018, lack of significant coherence between Niño3.4 and rainfall imply that increased rainfall variability is likely an effect of the IOD with influence from ENSO. At the sub-annual scale, significant coherence between the SST indices and rainfall is visible only during specific years and for short durations of time. Phase angles show that most of the time when correlations were highest, Niño3.4 SST and DMI were in phase with rainfall. During these times, rainfall variability is likely a result of SST variability in the Pacific (ENSO) and or Indian Ocean (IOD). However, we also observe some out of phase behavior between both SST indices and rainfall at sub-annual time scales. Out of phase implies that the two processes peak at different times. Rainfall variability during such times is likely caused by other factors separate from ENSO or IOD mode, or the influence of ENSO and IOD is modulated by other factors.

## Discussion

Given the importance of understanding localized patterns of rainfall variability in rainfed agropastoral systems, quantification of these patterns over space and time are needed. This work contributes to that need by characterizing spatial anomalies in wetting and drying periods over a 40-year time series of rainfall data and identifying domains of temporal variability that are expressed at interannual, interseasonal, and intraseasonal time scales. Importantly, regional patterns are not always consistent with patterns observed at the national scale, indicating the importance of multiscalar analyses to guide adaptation planning.

The SOM reveals archetypal patterns of rainfall anomalies and enhances our understanding of relative rainfall variability across space. Recurrent patterns are indicative of underlying climate drivers acting and interacting at multiple scales. Spatially coherent negative (Fig. 2, nodes 3,2) and positive (Fig. 2, nodes 1,1) anomalies at the national scale are most likely, largely a result of synoptic scale factors influencing rainfall across large areas of EA. Spatially heterogeneous patterns of negative and positive anomalies on the other hand likely result from interactions between large scale teleconnections and local level factors such as the influence of topography on local air flow and influence of water bodies on moisture availability and convective activity. Heterogeneous SOM patterns are consistent with previous studies that have shown spatial differences in rainfall characteristics across EA with spatial coherence varying among months of the same season^9,102^. In this study, we map the spatial patterns and identify their trends with time. The SOM patterns provide a basis for further investigation of the role of potential driving factors to better understand rainfall patterns. The trends of occurrence of identified patterns can be related to the increasing or decreasing roles of underlying factors and indicate the direction of change in rainfall for specific areas in space. For example, more than half the time, MAM and OND months between 1981 and 2021 have been dryer than average. While the frequencies of both anomalously dry and wet MAM months have been increasing over time, those of anomalously dry OND months have been decreasing. Overall, MAM is increasingly characterized by more months of higher or lower than average rainfall, while OND is increasingly characterized by more months of higher-than-average rainfall and less months of lower-than-average rainfall. More extreme MAM months imply poor rainfall distribution in the season and is unfavorable for rainfed agriculture and pastoral systems in arid and semi-arid areas. Adaption plans in such systems might consider strategies for dealing with prolonged dry periods in MAM and excess rainfall in OND.

While the SOM detects increasing MAM and OND extremes which is consistent with sub-seasonal variability, we characterize further the average periodicity of rainfall using wavelet analysis and detect variability at multiple time scales. Intermittent spells of significant intraseasonal and interseasonal variability become prominent after 1995, more strongly so after 2013 at the regional (Narok) level. Differences in the wavelet analysis results between levels of analysis suggest that averaging rainfall data over a larger geographic extent leads to loss of spatial variations of rainfall characteristics. This is an important result because local patterns are most relevant for place-based climate adaptation strategies such as stress resistant crop varieties^103^ and water harvesting for supplement irrigation^104^. This is especially true in arid agropastoral landscapes that are already vulnerable. In a bimodal rainfall regime like in Kenya, interseasonal variability suggests contrast between the rain season and the preceding or following dry season. Such alternating cycles of anomalously wet and dry seasons can lead to severe erosion or landscape degradation^105^ for fragile arid ecosystems.

We detect evidence of existing linkages between ENSO and IOD, and their individual and combined influence on EA rainfall variability. Consistent with existing literature that indicates greater influence on EA rainfall when ENSO and IOD are in phase^106^, the current study shows increased variability at specific times (e.g., 1997, 2006) when ENSO and IOD were in phase. However, independent occurrences of a positive (negative) IOD and El Niño (La Niña) have also been associated with anomalous rainfall in EA^107^, such as the extreme rainfall in 2019 that resulted from an independently occurring positive IOD mode. Coherence between Niño3.4 and Kenya rainfall at the annual time scale appears to have diminished after 2013, coinciding with increased rainfall variability that we observe in arid Narok. The increased variability after 2013 is likely a part of an abrupt climate shift that has been linked to climate change induced warming of Western Pacific SST^108,109^, and involving declining MAM rainfall and an enhanced link between OND La Niña events and subsequent dry MAM seasons^56^. The effect is increased contrast between seasons which we observe here as an increase in interseasonal variability. These results highlight shifting patterns of rainfall variability through time and enhance our understanding of the non-stationarity of teleconnections which are likely linked to climate change.

In general, our assessment shows that CHIRPS data conforms to those of spatial and aggregate monthly rainfall for EA, and that identified patterns are consistent with physical mechanisms that drive them. In this case, we note that wavelet spectra exhibit changes associated with the influence of ENSO and IOD. However, local analysis of rainfall patterns is hindered by a lack of reliable observations at fine spatial and temporal scales sufficient to characterize variability, thus one limitation of this study is a reliance on a single observed rainfall source, CHIRPS v2. As discussed earlier in the paper, multiple recent studies suggest that CHIRPS remains among the most reliable precipitation datasets for Kenya and has been widely used across the region. As also discussed, CHIRPS is still likely to have errors and localized assessments of skill have suggested that CHIRPS might struggle to capture the climates on Kenyan Highlands and on the shores of Lake Victoria. In future work, we plan to assess the sensitivity of our results to multiple gridded precipitation estimates that have performed well in reanalysis studies.

The numerous free parameters of the SOM approach can also introduce uncertainty in the interpretation of the SOM results^88^. However, spatial SOM patterns were largely consistent between the Narok and Kenya levels of analysis, suggesting our SOM parameters were sound and indicating potential generalization of this approach to other regions. Furthermore, sensitivity tests were performed on all the parameters and the best SOM chosen based on standard measures of SOM quality, discussed in detail in the methods section.

## Conclusions

In rainfall dependent agropastoral systems, unpredictability of rainfall patterns severely limits the ability of farmers and herders to plan and can undermine agricultural investment, negatively affecting economic development. This is especially true in East Africa, where multiscale climate drivers generate highly heterogeneous rainfall characteristics, yet adaptation plans are based on broad scale climate assessments. In this study, we characterize aspects of rainfall variability at scales relevant for place-based adaptation planning. We determine archetypal patterns of rainfall anomalies that demonstrate regional heterogeneity. These results enhance our understanding of local rainfall patterns and provide a basis for further investigation into local climate dynamics including potential microscale to mesoscale drivers of observed patterns and their changing roles. In addition, we determine the temporal timescales of rainfall variability and how it is changing over time and establish that a localized assessment can better highlight features that are not readily discernible at broader scales. We elaborate on subtle patterns of rainfall variability observed at the annual, seasonal, and sub-seasonal scale which relates to inconsistent rainfall distribution that we refer here as intraseasonal variability. Improved understanding of localized rainfall patterns is necessary for contextualized adaptation strategies such as agricultural climate services. It further highlights relative vulnerabilities associated with spatial heterogeneity of rainfall and this can help to focus resources where they are most needed and guide the transfer of successful adaptation strategies across spatially coherent areas. This information can be integrated into national adaptation planning to support effective place-based strategies that address actual local needs, for example though a decision support tool to guide targeted recommendations. Furthermore, the SOM patterns can help to define spatial scales associated with anomalous occurrences of aridity or wetness which is useful for defining spatial domains of early warning systems. While Narok was used as a case study site for regional level analysis because it captures the wide range of conditions present in Kenya, results for other regions are likely to be locally specific.

### Supplementary Information

Supplementary Figures.

## Data Availability

All the data used in this analysis are publicly available. The Climate Hazards group InfraRed Rainfall with Station (CHIRPS) v2.0 dataset is available from https://www.chc.ucsb.edu/data/chirps. Monthly DMI and Niño3.4 SST anomalies are available from https://psl.noaa.gov/gcos_wgsp/Timeseries/. Code is available from the corresponding author upon request.
