# Supplementary material for: Observations of enhanced rainfall variability in Kenya, East Africa
Source: Sci Rep. 2024 Jun 5;14:12915. doi: 10.1038/s41598-024-63786-2 (PMC11153539; doi:10.1038/s41598-024-63786-2)
Supplement: Supplementary file 1 — Supplementary Figures. [file 41598_2024_63786_MOESM1_ESM.docx]

**Supplementary information**


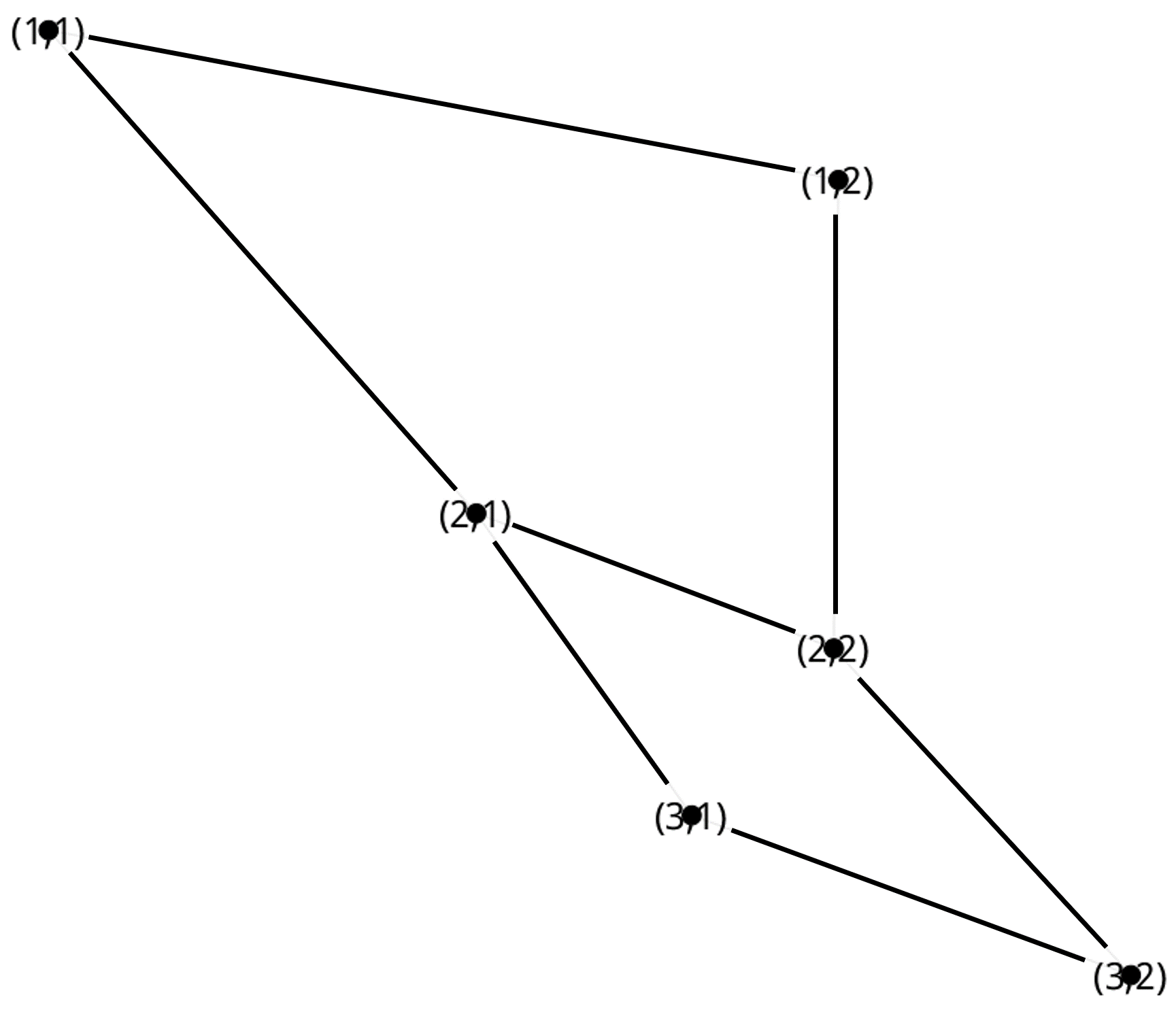


**Supplementary Fig 1:** Sammon distortion map representing the relative Euclidean distances between the Kenya MAM SOM nodes shown in Fig. 2a. The Sammon map is well ordered with no overlapping nodes. Nodes that are further apart are more differentiated than those that are closer together.


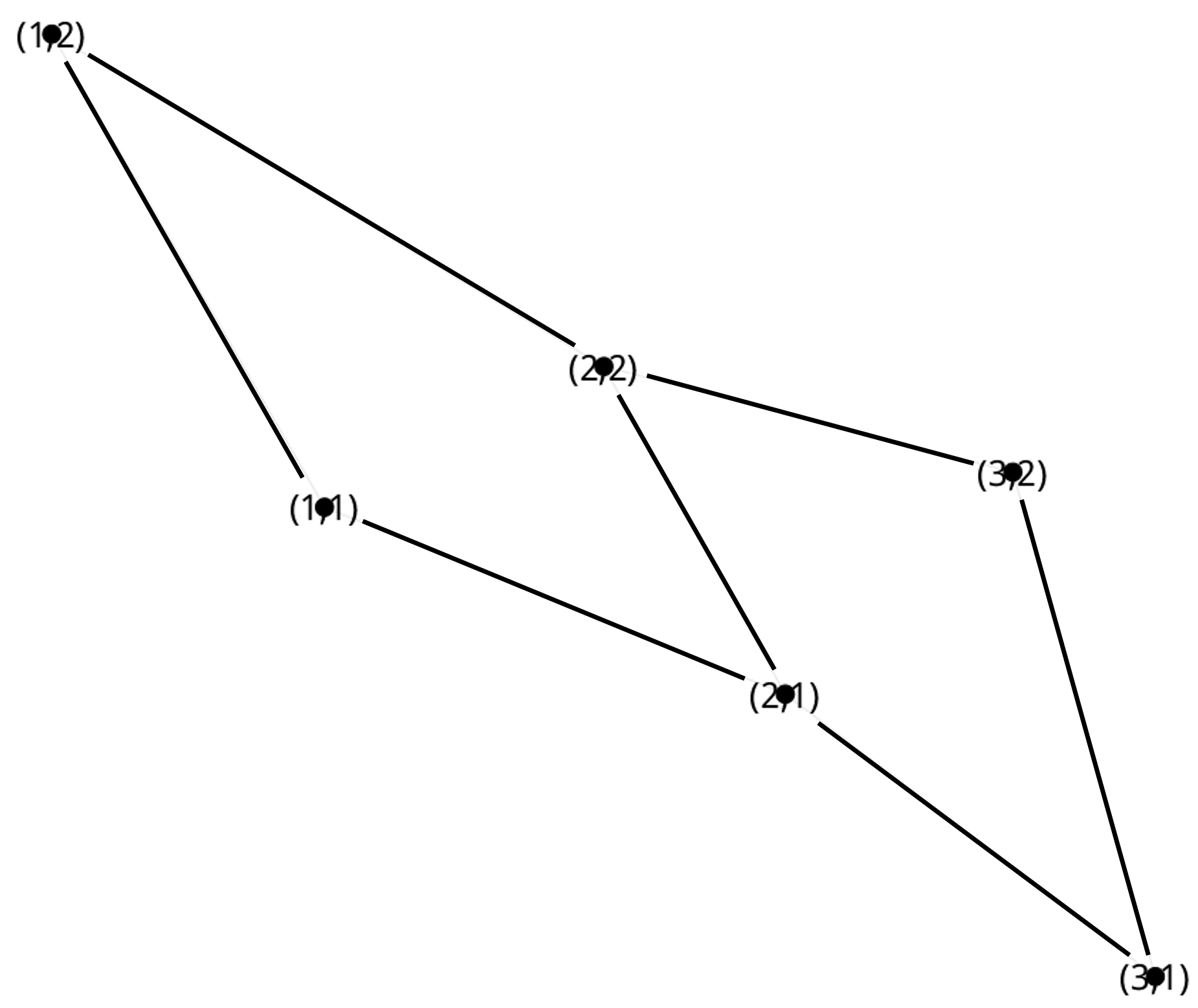


**Supplementary Fig 2:** Sammon distortion map representing the relative Euclidean distances between the Kenya OND SOM nodes shown in Fig. 2b. The Sammon map is well ordered with no overlapping nodes. Nodes that are further apart are more differentiated than those that are closer together.


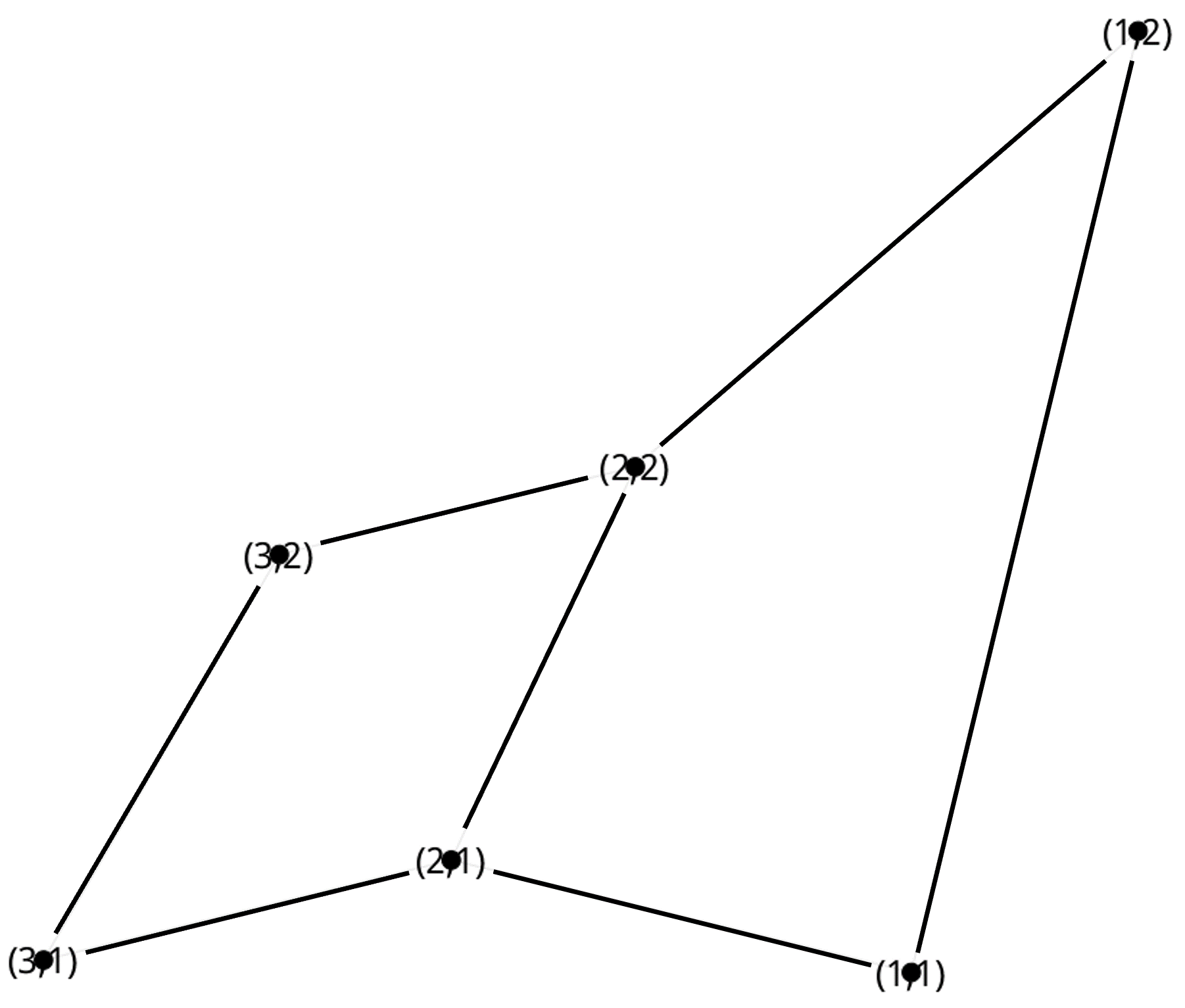


**Supplementary Fig 3:** Sammon distortion map representing the relative Euclidean distances between the Narok MAM SOM nodes shown in Fig. 3a. The Sammon map is well ordered with no overlapping nodes. Nodes that are further apart are more differentiated than those that are closer together.


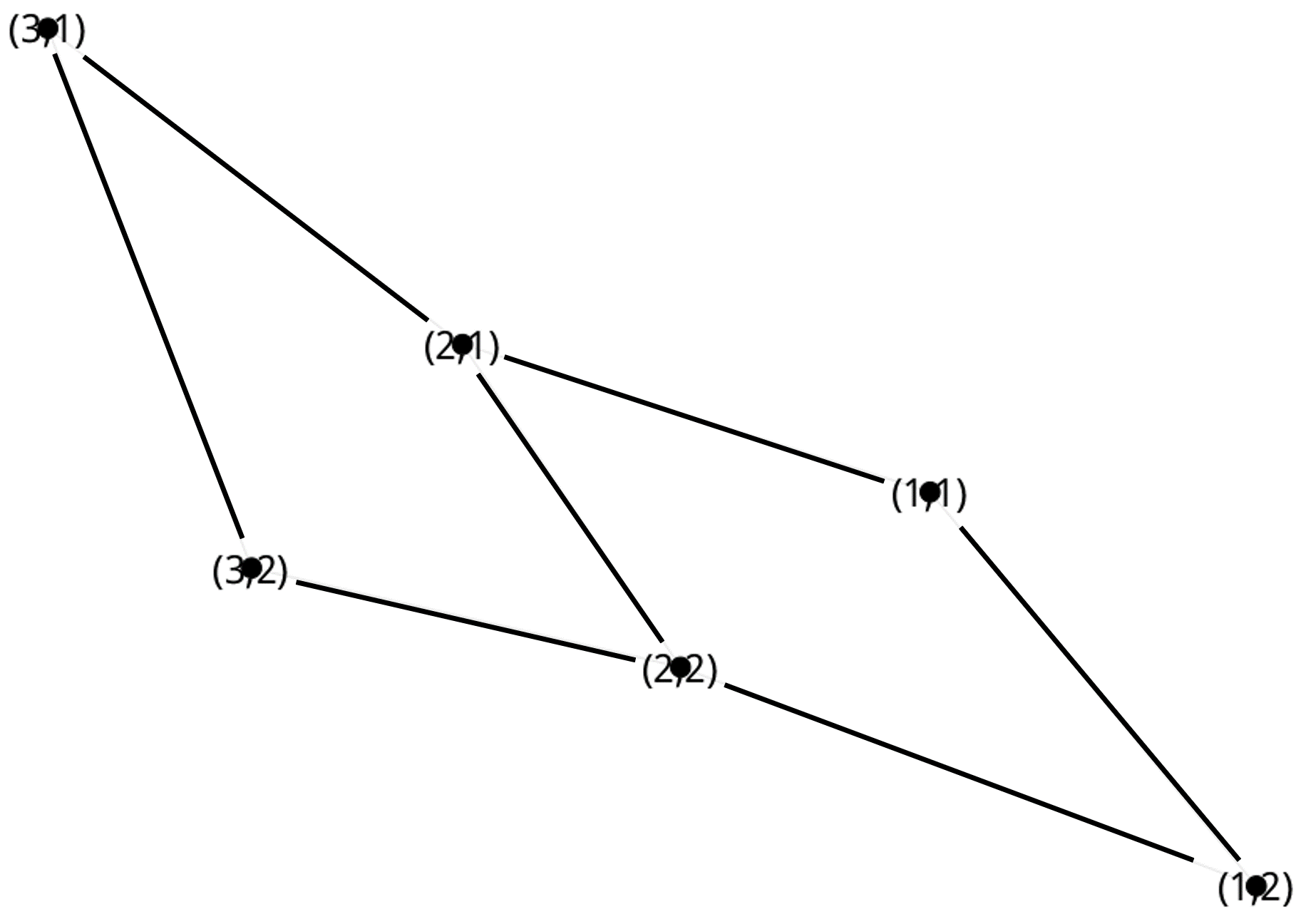


**Supplementary Fig 4:** Sammon distortion map representing the relative Euclidean distances between the Narok OND SOM nodes shown in Fig. 3b. The Sammon map is well ordered with no overlapping nodes. Nodes that are further apart are more differentiated than those that are closer together.

**
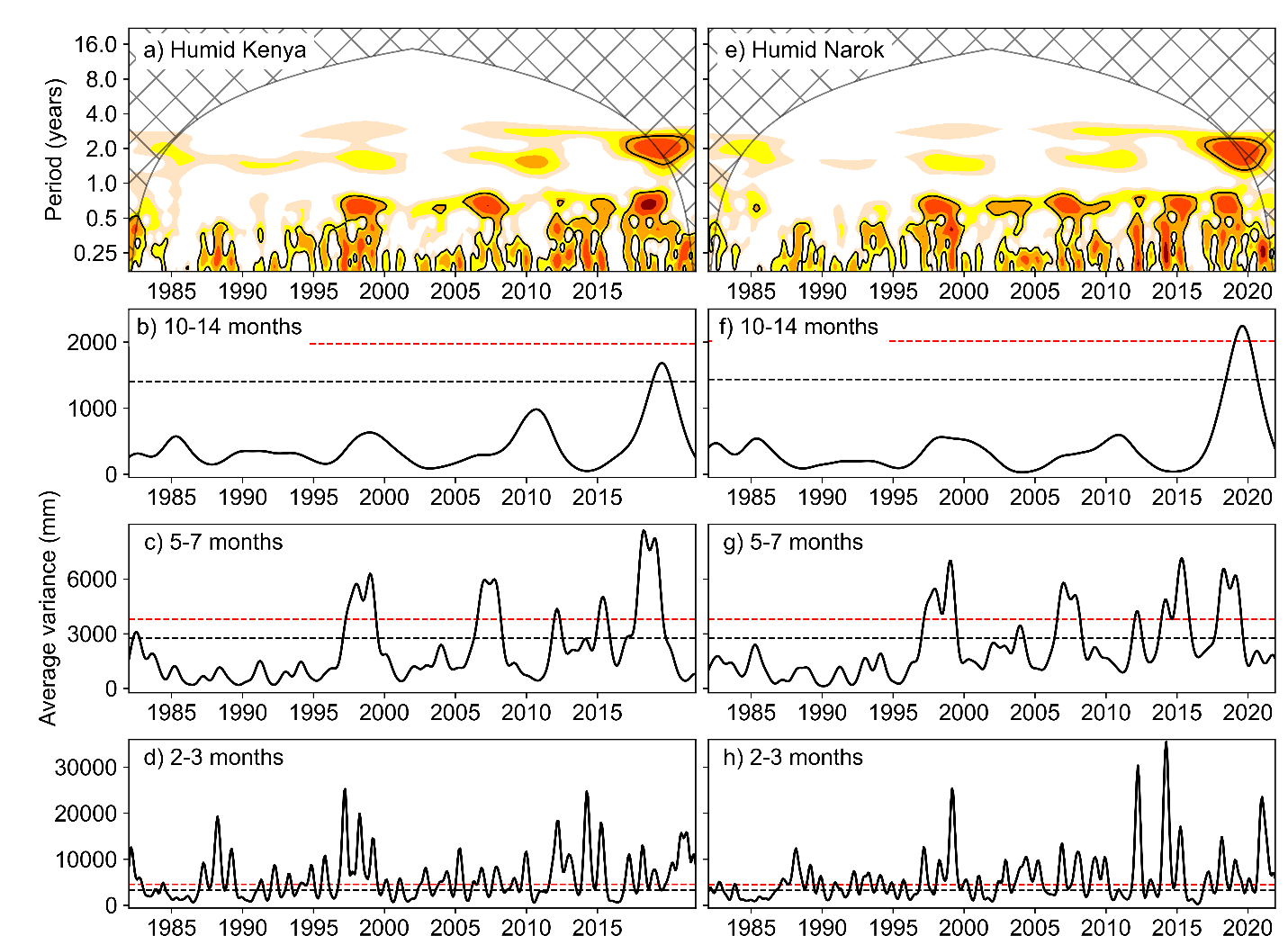
**

**Supplementary Fig 5:** Wavelet power spectra of spatially averaged rainfall over humid agro-climatic zones of Kenya (a) and Narok (e). In a) and e), color contours represent wavelet power normalized by the squared standard deviation and are at 0, 1, 2, 3, 4 from the lightest to the darkest colors, solid black contours enclose areas of >0.95 significance relative to a lag-1 red noise spectrum, hatched areas represent the cone of influence – area of the wavelet spectrum where edge effects (errors) due to a finite time series cannot be ignored. Figures b), c), d) and f), g), h), respectively show scale averaged power over the periods 18-30, 5-10, and 2-4 months for a) and e), and show average rainfall variance for those periods. Dotted horizontal lines show the >0.95 (black) and >0.99 (red) significance levels. Curves above the dotted lines have significant variance.
